# Gastrointestinal adverse events with combination of checkpoint inhibitors in advanced melanoma: a systematic review

**DOI:** 10.2217/mmt-2017-0027

**Published:** 2018-01-18

**Authors:** Elizabeth S Mearns, Jill A Bell, Aaron Galaznik, Stefanie M Puglielli, Allie B Cichewicz, Talia Boulanger, Ignacio Garcia-Ribas

**Affiliations:** 1 Truven Health Analytics, an IBM Company, 75 Binney Street, Cambridge, MA 02142, USA; 2 Millennium Pharmaceuticals, Inc., a wholly owned subsidiary of Takeda Pharmaceutical Company Limited, 40 Landsdowne Street, Cambridge, MA 02139, USA

**Keywords:** advanced melanoma, anti-CTLA-4, anti-PD-1, checkpoint inhibitors, gastrointestinal adverse events, ipilimumab, nivolumab, systematic literature review

## Abstract

**Introduction:**

Immunotherapies, including checkpoint inhibitors (CIs) such as cytotoxic T-lymphocyte antigen-4 (CTLA-4) and programmed death-1 (PD-1) inhibitors, are revolutionizing the treatment of advanced melanoma. Combining CTLA-4 and PD-1 inhibitors provides additional clinical benefit compared with single agents alone. However, combination therapy can increase the incidence of gastrointestinal adverse events (GI AEs). This systematic review assessed the epidemiological, clinical, economic, and humanistic burden of GI AEs due to combination CIs in advanced melanoma.

**Methods:**

MEDLINE, EMBASE, and the Cochrane Library were systematically searched (December 2011 to December 2016) to identify primary studies, systematic reviews, meta-analyses, and conference proceedings (2014–2016) evaluating adults treated with ≥2 CIs for advanced melanoma.

**Results:**

Of the 3391 identified articles, 14 were included. Most studies examined the ipilimumab plus nivolumab combination. Any grade and grade 3–4 GI AEs occurred in more patients receiving ipilimumab plus nivolumab versus ipilimumab or nivolumab alone. The most common grade 3–4 GI AEs were diarrhea and colitis. Grade 3–4 colitis occurred in more patients receiving ipilimumab plus nivolumab. However, grade 3–4 diarrhea occurred at the same rate as ipilimumab alone. GI AEs developed with ipilimumab plus nivolumab approximately 6.6 weeks after initiating treatment. No studies assessing the economic or humanistic burden of GI AEs were identified.

**Conclusion:**

GI AEs occurred at a higher rate and greater severity in patients treated with ipilimumab plus nivolumab versus ipilimumab or nivolumab monotherapy. The lack of research on economic and humanistic burden of GI AEs with combination CIs for advanced melanoma represents an unmet need in the literature and should be explored in future studies.

In 2016, the National Cancer Institute estimated that there would be 76,380 new cases of melanoma and approximately 10,130 patients would die from the disease that year in the USA alone [1]. First-line therapies for advanced unresectable or metastatic melanoma include immunotherapy and targeted therapy, while chemotherapy is withheld for bridging or as a second-line option [2–4]. The introduction of targeted therapies such as BRAF and MEK inhibitors (e.g., vemurafenib, encorafenib and dabrafenib used alone or in combination with binimetinib, cobimetinib and trametinib) in recent years has substantially improved the treatment success for patients with advanced melanoma, offering high overall response rates (ORR) and progression-free survival (PFS). Immunotherapies that utilize antibodies to bind checkpoint inhibitors of T cells (termed checkpoint inhibitors) have also demonstrated remarkable efficacy in the treatment of advanced melanoma [5]. In clinical trials, both anti-cytotoxic T lymphocyte-associated protein 4 (CTLA-4; e.g., ipilimumab) and anti-programed cell death-1 (PD-1) agents (e.g., nivolumab, pembrolizumab) have demonstrated improved overall survival, PFS and ORR in patients with advanced melanoma compared with chemotherapy [6–11]. Following the availability of evidence that ipilimumab plus nivolumab significantly improved ORR and PFS compared with ipilimumab alone, the ipilimumab–nivolumab combination was approved for unresectable, advanced melanoma [12].

Similar to the immune mechanism of antitumor activity, the checkpoint inhibitors are associated with a high frequency of immune-related adverse events (AEs). The dual checkpoint blockade with ipilimumab plus nivolumab has produced more serious AEs as well as a higher incidence of AEs compared with single-agent therapy [13]. These AEs can affect a variety of organ systems [14,15]. Many treatment-related AEs with ipilimumab are severe and in some cases can be fatal [14]. Most notably, severe AEs of the gastrointestinal (GI) tract such as colitis may lead to GI perforation and associated mortality.

We performed a systematic literature review to evaluate the epidemiological, clinical, humanistic and economic burden of GI AEs associated with combination checkpoint inhibitors in adults with advanced, unresectable melanoma. Rates of GI AEs associated with single-agent therapies evaluated within these studies of combination therapies were included for comparison.

## Methods

A systematic review of MEDLINE, EMBASE and the Cochrane Library identified published studies (from December 2011 to December 2016) assessing economic, clinical, humanistic or epidemiological burden of AEs with combination checkpoint inhibitors in advanced melanoma. Searches identified literature published in English during the last 5 years and excluded case reports and narrative reviews. The search strategy combined the medical subject heading terms and keywords for melanoma and checkpoint inhibitors (Table 1). Manual backwards citation tracking of references from included studies and review articles was performed to identify additional relevant studies. Searches were also performed in abstracts presented at the American Society of Clinical Oncology, European Society of Medical Oncology and International Society for Pharmacoeconomics and Outcomes Research conferences between 2014 and 2016. One reviewer screened the search yield to select a list of titles to be considered as sources for the review. Two reviewers screened the potential full-text citations independently with discrepancies resolved by a third reviewer. Two reviewers abstracted all data with disagreements resolved by discussion or a third reviewer.

**Table 1.** **Search in MEDLINE (PubMed).**

**Search No.**	**Search terms**
1	Pembrolizumab[Title/Abstract] or nivolumab[Title/Abstract] or pidilizumab[Title/Abstract] or ipilimumab[Title/Abstract] or tremelimumab[Title/Abstract] or durvalumab[Title/Abstract] or MEDI4736[Title/Abstract] or atezolizumab[Title/Abstract] or MPDL3280A[Title/Abstract] or avelumab[Title/Abstract] or MSB0010718C[Title/Abstract] or pd-1 inhibitor*[Title/Abstract] or pd-L1 inhibitor*[Title/Abstract] or CTLA-4 inhibitor*[Title/Abstract] or “anti-pd-1”[Title/Abstract] or “anti-pd-L1”[Title/Abstract] or “anti-ctla-4”[Title/Abstract] or checkpoint inhibitor*[Title/Abstract] or “pd-1/pd-L1”[Title/Abstract]

2	Melanoma[MeSH Terms] or melanoma*[Title/Abstract]

3	#1 and #2

4	Case reports[pt]

5	#3 not #4

6	Review[pt] not (systematic or Cochrane or meta-analy*)

7	#5 not #6

8	#7 and Filters: published in the last 5 years

MeSH: Medical Subject Heading.

To be included, studies had to: be published in English within the last 5 years; be a primary study, systematic review or meta-analysis in adults with advanced melanoma; treat patients with combination (more than or equal to two) of checkpoint inhibitor therapy (e.g., PD-1, PD-L1, CTLA-4) in at least one arm of the study; and report the economic, clinical, humanistic or epidemiological burden of GI AEs attributed to checkpoint inhibitor therapy. Studies assessing patients with uveal or ocular melanoma were not included in this literature review. The primary focus of this systematic review was on the combined use of two or more checkpoint inhibitors; single-agent checkpoint inhibitor therapies were assessed as comparators in included studies, but were not systematically evaluated.

## Results

### Study characteristics

**Figure 1.**
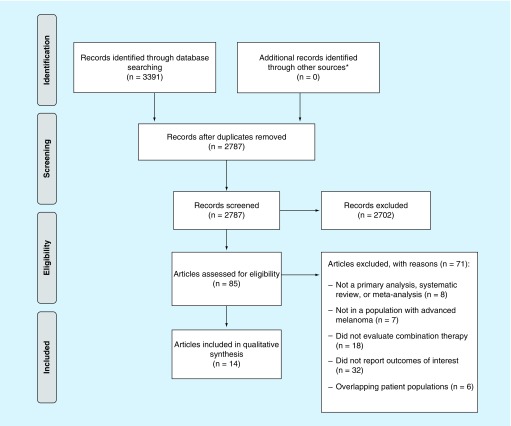
**Flow diagram of literature search results.** *Additional searches included manual backwards citation tracking and conference abstracts presented at the American Society of Clinical Oncology, European Society of Medical Oncology and the International Society for Pharmacoeconomics and Outcomes Research.

The searches identified 3391 citations with no additional citations identified through backwards citation tracking (Figure 1). After removal of duplicates and title/abstract screening, 85 were eligible for full-text screening, and 14 reports (11 principal studies and 3 updated analyses or subgroup analyses) were included in the systematic review (Table 2) [7,16–28]. Six reports were clinical studies, five were retrospective analyses and three were meta-analyses (two network and one traditional). Of the 11 principal studies, all assessed epidemiological burden and most assessed clinical burden (n = 7). No studies were found on the humanistic or economic burden of GI AEs.

**Table 2.** **Summary of studies included.**

**Study (year)**	**n**	**Study design**	**Country**	**Interventions evaluated**		**Type of burden**	**Ref.**

				**Combination**	**Comparator**		
Abdel-Rahman *et al*. (2016)	1140	Systematic review^†^ and meta-analysis	Multinational	IPI + NIVO	IPI	EPI	[16]

Altman *et al*. (2016)	85	Retrospective, single-center analysis	USA	Anti-CTLA-4 + anti-PD-1	Anti-CTLA-4 or anti-PD-1	EPI, clinical	[26]

Chapman *et al*. (2016)	252	CheckMate 218: expanded access program	Canada, USA	IPI + NIVO	NA	EPI, clinical	[28]

CiRen *et al*. (2016)	6442	Systematic review^†^ and meta-analysis	Multinational	IPI + NIVO	IPI or NIVO	EPI	[17]

Friedman *et al*. (2016)	64	CheckMate 218: Single-center analysis of expanded access program	Canada, USA	IPI + NIVO	NA	Clinical	[22]

Friedman *et al*. (2016)	106	Retrospective, single-center analysis	USA	IPI + NIVO	IPI, anti-PD-1	EPI, clinical	[25]

Hodi *et al*. (2016)	142	CheckMate 069: 2-year follow-up	France, USA	IPI + NIVO	IPI	EPI	[20]

Postow *et al*. (2015)	142	CheckMate 069: Phase II clinical trial	France, USA	IPI + NIVO	IPI	EPI^‡^, clinical	[19]

Larkin *et al*. (2015)	945	CheckMate 067: Phase III clinical trial	Multinational	IPI + NIVO	IPI or NIVO	EPI, clinical	[7]

Spain *et al*. (2016)	353	Retrospective, single-center analysis	UK	IPI + NIVO	IPI or anti-PD-1	EPI, clinical	[24]

Sznol *et al*. (2016) Sznol *et al*. (2016)	448	CheckMate 067, CheckMate 069, CA209–004: pooled analysis of three clinical trials	Multinational	IPI + NIVO	NA	EPI, clinical	[21,23]

Wolchok *et al*. (2013)	86	Phase I clinical trial	USA	IPI + NIVO	IPI followed by NIVO	EPI	[18]

Yang *et al*. (2016)	6405	Network meta-analysis of 19 studies	Multinational	IPI + NIVO	IPI, PEMBRO, NIVO, TREM, chemo, or IPI + chemo	EPI	[27]

^†^Data from this systematic review were not incorporated into our analysis since the primary studies evaluated were already included.

^‡^Epidemiological data were taken from the 2-year follow-up (Hodi *et al*., 2016) since it reported the most recent numbers [20].

Anti-CTLA-4: Anti-cytotoxic T lymphocyte-associated molecule-4; EPI: Epidemiological; IPI: Ipilimumab; NA: Not applicable; NIVO: Nivolumab; PEMBRO: Pembrolizumab; TREM: Tremelimumab.

Most studies evaluated the ipilimumab plus nivolumab combination (n = 11); however, one did not specify treatments and only reported that patients were treated with anti-PD-1 and anti-CTLA-4 monoclonal antibodies [26]. Among the studies included in this review, treatment duration ranged from 11 to 19 months, or until disease progression or unacceptable toxicity, and median follow-up ranged from 1 to 24.5 months. Between 25 and 68% of patients were males, and median age ranged from 58 to 65 years. Patients came from various geographical regions; clinical studies took place in up to 22 countries within North America, Europe, Australia and the Middle East, and retrospective studies took place within the USA or the UK. Of studies that reported AE grading systems, all AEs were graded according to the Common Terminology Criteria for Adverse Events (CTCAE), version 3.0 or 4.0 (Table 3) [29].

**Table 3.** **Criteria for grading of gastrointestinal adverse event severity.**

**Adverse event**	**Grade 1**	**Grade 2**	**Grade 3**
Diarrhea	Increase of less than four stools per day over baseline; mild increase in ostomy output compared with baseline	Increase of four to six stools per day over baseline; moderate increase in ostomy output compared with baseline	Increase of more than or equal to seven stools per day over baseline; incontinence; hospitalization indicated; severe increase in ostomy output compared with baseline; limiting self care ADL

Colitis	Asymptomatic; clinical or diagnostic observations only; intervention not indicated	Abdominal pain; mucus or blood in stool	Severe abdominal pain; change in bowel habits; medical intervention indicated; peritoneal signs

Nausea	Loss of appetite without alteration in eating habits	Oral intake decreased without significant weight loss, dehydration or malnutrition	Inadequate oral caloric or fluid intake; tube feeding, TPN or hospitalization indicated

Vomiting	One to two episodes (separated by 5 min) in 24 h	Three to five episodes (separated by 5 min) in 24 h	More than or equal to six episodes (separated by 5 min) in 24 h; tube feeding, TPN or hospitalization indicated

Abdominal pain	Mild pain	Moderate pain; limiting instrumental ADL	Severe pain; limiting self care ADL

Constipation	Occasional or intermittent symptoms; occasional use of stool softeners, laxatives, dietary modification or enema	Persistent symptoms with regular use of laxatives or enemas; limiting instrumental ADL	Obstipation with manual evacuation indicated; limiting self care ADL

Enterocolitis	Asymptomatic; clinical or diagnostic observations only; intervention not indicated	Abdominal pain; mucus or blood in stool	Severe or persistent abdominal pain; fever; ileus; peritoneal signs

Grade 4 = Life-threatening consequences; urgent intervention indicated; Grade 5 = Death.

Adapted from Common Terminology Criteria for Adverse Events v4.0.

ADL: Activities of daily living; TPN: Total parenteral nutrition.

### Epidemiological burden

#### Incidence of GI AEs

Patients treated with ipilimumab plus nivolumab experience more GI AEs compared with patients treated with ipilimumab or nivolumab alone. Of patients treated with ipilimumab plus nivolumab, 38.0–51.1% experienced a treatment-related GI AE while on treatment (Table 4) [7,18,19]. This incidence was higher than in patients receiving ipilimumab monotherapy (36.7–37.0%) or nivolumab monotherapy (19.5%) [7,18,19]. A similar trend was observed with grade 3–4 treatment-related GI AEs (ipilimumab plus nivolumab, 9.0–21.3%; ipilimu mab, 10.9–11.6%; nivolumab, 2.2%), although the incidence of severe AEs with ipilimumab therapy does overlap with the lower end of the range for the ipilimumab–nivolumab combination [7,18,19,28].

**Table 4.** **Incidence of treatment-related gastrointestinal adverse events.**

**Treatment-related AEs**	**Any grade**			**Grade 3–4**			**Ref.**

	**IPI + NIVO (%)**	**IPI (%)**	**NIVO (%)**	**IPI + NIVO (%)**	**IPI (%)**	**NIVO (%)**	
Any GI event	38.0–51.1	36.7–37.0	19.5	9.0–21.3	10.9–11.6	2.2	[7,18,19,28]

Diarrhea	34.0–45.0	33.1–35.0	19.2	6.0–10.0	6.1–11.0	2.2	[7,18,20]

Colitis	9.0–18.0	7.0–11.6	1.3	4.0–13.0	2.0–8.7	0.6	[7,18,20]

Nausea	21.0–25.9	16.1–20.0	13.1	1.0–2.2	0.6–2.0	0	[7,20]

Decreased appetite	12.0–17.9	9.0–12.5	10.9	0–1.3	0–0.3	0	[7,20]

Vomiting	13.0–15.3	7.0–7.4	6.4	1.0–2.6	0–0.3	0.3	[7,20]

Abdominal pain	13.0	11.0	–	0	2.0	–	[20]

Constipation	9.0	0	–	1.0	0	–	[20]

Enterocolitis	1.1	0	–	1.1	0	–	[19]

Incidence of AEs based on the following treatment regimens: IPI 3 mg/kg plus NIVO 1 mg/kg intravenously every 3 weeks for four doses, followed by NIVO 3 mg/kg intravenously every 2 weeks; IPI 3 mg/kg intravenously every 3 weeks for four doses; NIVO 3 mg/kg intravenously every 2 weeks for four doses.

AE: Adverse event; GI: Gastrointestinal; IPI: Ipilimumab; NIVO: Nivolumab.

Data taken from [7,18–20,28].

##### Colitis

Colitis occurred in more patients with the ipilimumab–nivolumab combination, and cases were mostly severe. Compared with ipilimumab (7.0–11.6%) and nivolumab (1.3%) monotherapy, the incidence of any grade of treatment-related colitis was highest with these drugs in combination (9.0–18.0%) [7,18,20]. The same was true with grade 3–4 treatment-related colitis; its incidence with the combination ranged from 4.0 to 13.0%, overlapping with, but greater than, the range observed with ipilimumab alone (2.0–8.7%) [7,18,20]. Matching the pattern seen for treatment-related GI AEs overall, both any-grade and grade 3–4 treatment-related colitis occurred less often with nivolumab monotherapy [7]. While treatment-related colitis was uncommon, most patients who developed colitis with ipilimumab plus nivolumab developed severe cases. In a pooled analysis of trial data, the incidence was 13.0% for any grade of colitis and 9.0% for grade 3–4 colitis, suggesting that approximately 70% of colitis cases with combination therapy are severe or life threatening [23]. One study found that grade 3–4 enterocolitis, a subtype of colitis that only affects the small intestine and colon, occurred in only 1.1% of patients treated with ipilimumab plus nivolumab, and was not reported in any patients receiving ipilimumab alone [19].

##### Diarrhea

Among all studies, treatment-related diarrhea was the most common GI AE, occurring in nearly half of patients treated with the ipilimumab–nivolumab combination. The incidence of any grade of treatment-related diarrhea with the combination therapy overlapped with, but exceeded, that of ipilimumab monotherapy [7,18,20]. The incidence of any grade of treatment-related diarrhea with nivolumab monotherapy was lower (19.2%) than both combination therapy (34.0–45.0%) and ipilimumab monotherapy (33.1–35.0%) [7].

The incidence of grade 3–4 diarrhea in patients treated with ipilimumab plus nivolumab was low. Treatment-related grade 3–4 diarrhea was rare, and similar, between combination therapy (6–10%) and ipilimumab monotherapy (6–11%). Following the general trend among other comparisons with nivolumab, the frequency of diarrhea was even lower with nivolumab monotherapy (2.2%) [7].

##### Nausea

Treatment-related nausea was experienced by less than a quarter of patients. The incidence of any grade of treatment-related nausea followed the same pattern as other GI AEs, with overlapping, but higher incidence of nausea with the combination therapy (21.0–25.9%) than with ipilimumab monotherapy (16.1–20.0%), and the lowest incidence with nivolumab monotherapy (13.1%) [7,20]. Grade 3–4 treatment-related nausea did not appear to differ among these treatments and occurred infrequently (0–2.2%).

##### Other treatment-related GI AEs

Other treatment-related GI AEs occurred less frequently than colitis, diarrhea and nausea, and with very few severe cases. Of these, any grade of appetite loss was the most common across all treatment groups, but occurred most frequently with ipilimumab plus nivolumab (12.0–17.9%) compared with ipilimumab (9.0–12.5%) or nivolumab (10.9%) alone [7,20]. Any grade of abdominal pain and constipation with the ipilimumab plus nivolumab were reported in one Phase II clinical trial and affected <15% of patients (n = 94) [19,20]. Grade 3–4 treatment-related AEs for all GI disorders other than colitis and diarrhea occurred infrequently; AEs ranged from 0 to 2.6% with no major differences between combination therapy or monotherapies. Constipation (any grade and grade 3–4) was only experienced by patients treated with the ipilimumab–nivolumab combination, and did not occur at all in those treated with ipilimumab monotherapy (0%) [20]. Therefore, while severe treatment-related diarrhea and colitis were a common problem with the ipilimumab–nivolumab combination and ipilimumab monotherapy, other treatment-related GI AEs appeared to be mild.

#### Risk of GI AEs

The risk of any grade of diarrhea with the combination was examined in two network meta-analyses, which found a significantly higher risk with the combination when compared with ipilimumab monotherapy, nivolumab monotherapy, pembrolizumab monotherapy or chemotherapy [17,27]. However, a traditional meta-analysis found no significant difference between the ipilimumab-combination and ipilimumab monotherapy in the relative risk (RR) of grade 3–5 diarrhea (RR: 1.38; 95% CI: 0.85–2.25; I2 = 0%) or colitis (RR: 1.34; 95% CI: 0.47–3.80; I2 = 64%) [16]. Similarly, one of the two network meta-analyses found no significant difference in the risk of nausea with the combination compared with checkpoint inhibitor monotherapy, chemotherapy or ipilimumab plus chemotherapy [27].

### Clinical burden

#### Timing & severity of GI AEs

GI AEs in patients receiving the ipilimumab–nivolumab combination have been reported to begin during the second month of therapy and can be severe enough to cause treatment discontinuation. In one clinical study, the median time to GI AEs was 6.6 weeks after patients began receiving the ipilimumab–nivolumab combination [28]. A pooled analysis of three clinical trials reported that eight percent of patients discontinued the combination due to diarrhea and seven percent due to colitis [23]. Further, one trial reported higher discontinuation due to diarrhea or colitis with the combination (diarrhea: 8.3%; colitis: 8.3%) than with ipilimumab (diarrhea: 4.5%; colitis: 7.7%) or nivolumab monotherapy (diarrhea: 1.9%; colitis: 0.6%) [7].

Death due to GI AEs appears to be rare, but in one trial, a patient treated with the combination died due to a delay in the treatment of immune colitis, leading to sepsis and multiorgan failure [21]. No deaths due to GI AEs with checkpoint inhibitor monotherapy were reported.

#### Treatment of GI AEs

Due to the recency of combination therapy with checkpoint inhibitors, treatment and management of GI AEs were not comprehensively reported in the literature. Of the studies that did report pharmacologic management of GI AEs, most of the patients treated with ipilimumab plus nivolumab required immunomodulatory agents (IMAs; e.g., corticosteroids or the anti-TNF-α, infliximab) for the treatment of lower GI AEs (e.g., colitis and diarrhea), most of which resolved in just over a month. In two clinical trials, approximately 49–65% of patients using the ipilimumab–nivolumab combination received IMAs for GI AEs, similar to the percentage observed in patients using ipilimumab alone (47–65%) but higher than in those using nivolumab alone (14.8%) [7,19]. Patients with the combination received IMAs for grade 3–4 AEs at a lower rate (85–89%) than with ipilimumab monotherapy (92–100%), but at a higher rate compared with nivolumab monotherapy (71%). GI AEs resolved with IMAs in most patients receiving the combination (any grade AE: 93–94%; grade 3–4 AE: 88–98%), slightly more than with ipilimumab (any grade: 78–88%; grade 3–4: 80–89%) or nivolumab (any grade AE: 71%; grade 3–4 AE: 50%) monotherapy. Any-grade GI AEs had a slightly longer median time to resolution with IMAs (4.5–4.7 weeks) than grade 3–4 GI AEs (3–4.3 weeks). These resolution rates with combination therapy were comparable to those observed with ipilimumab monotherapy (any grade AE: 4.9–5 weeks; grade 3–4 AE: 3.6–4.7 weeks). Resolution rates in patients with nivolumab were not evaluable.

Infliximab use for GI AEs in patients treated with the ipilimumab–nivolumab combination varies dramatically, with rates as low as 6% and as high as 38%. A pooled analysis of three clinical trials reported that six percent of patients treated with the combination were administered infliximab for GI AEs [21]. One clinical study and one retrospective analysis in elderly patients reported that infliximab was administered for diarrhea in 22–38% of patients treated with the combination [22,25]. The retrospective analysis also reported lower infliximab use for diarrhea in patients receiving ipilimumab (7%) or anti-PD-1 (0%) monotherapy compared with the combination regimen (38%) [25].

## Discussion

While there have been many systematic literature reviews on the burden of GI AEs with checkpoint inhibitor monotherapy for the treatment of advanced melanoma, to our knowledge, this is the first that focuses on treatment with combination checkpoint inhibitors. While various AEs occur with this drug class, we aimed to specifically assess the epidemiological, clinical, humanistic and economic burden of GI AEs. The studies identified for our review evaluated the epidemiological and clinical burden of AEs with use of the ipilimumab–nivolumab combination. The GI AE burden with other combinations of checkpoint inhibitors has not been well studied to date; similarly, the humanistic and economic burden of GI AEs with combination therapy remains largely uninvestigated.

Approximately half of the patients experience any grade of treatment-related GI AEs, with grade 3–4 occurring in up to 21.3% of patients [7,18,19,28]. Treatment-related colitis and diarrhea were among the most common of these. Diarrhea occurred more often than colitis; however, colitis tended to be more severe. It should be noted that while the Common Terminology Criteria for Adverse Events treats colitis and diarrhea as separate entities, they may not truly be clinically distinct and there is always the potential for overlap in reporting.

Across the categories of treatment-related GI AEs, the combination of ipilimumab and nivolumab consistently produced more AEs than either agent used alone. While incidence of these AEs tended to overlap with that of ipilimumab monotherapy, it ranged much higher. GI AEs were also consistently lowest with nivolumab monotherapy compared with either the ipilimumab–nivolumab combination or ipilimumab monotherapy. While this was an important difference, it must be regarded in perspective, as all studies reported that AEs with the ipilimumab–nivolumab combination remained manageable [7,18,20].

Further, in the published literature there are not many studies directly comparing incidence of GI AEs with combination treatment and single agents alone; there were only two studies comparing to ipilimumab monotherapy [7,20] and only one to nivolumab monotherapy [7]. Despite this limited set of direct comparisons, the incidence of AEs generally matched those previously reported in Phase III trials with these agents used alone [6,9].

Two network meta-analyses reported that the risk of developing diarrhea with combination therapy was higher than with chemotherapy or with monotherapy using ipilimumab, nivolumab or pembrolizumab [17,27]. Matching this pattern observed for risk of diarrhea, the incidence of diarrhea was also higher with combination treatment compared with ipilimumab or nivolumab monotherapy. This may suggest that, similarly, the incidence of diarrhea would be higher among patients using combination therapy compared with those given pembrolizumab monotherapy or chemotherapy. However, direct comparisons were not available to assess diarrhea incidence with either pembrolizumab or chemotherapy. Although a comprehensive review of the management of the immune-related GI AEs resulting from combination checkpoint inhibitors is outside the scope of this systematic review, we thought it was prudent to address the little that has been published on the management and treatment. The label for nivolumab directs the care of patients with serious GI AEs resulting from use of combination checkpoint inhibitor therapy. For serious cases of diarrhea or colitis with ipilimumab plus nivolumab, the nivolumab label recommends that treatment should be withheld or discontinued, and corticosteroids should be administered. For grade 2–3 colitis, treatment should be withheld, and for grade 4 or recurrent cases, it should be permanently discontinued [13]. Corticosteroids should be administered and then tapered, unless worsening or no improvement occurs, at which point the dose should be increased.

Corticosteroids were likely to have been used as recommended within the studies captured within the current review, which reported use of immunomodulatory agents for combination checkpoint inhibitor-induced GI AEs. While most studies did not specify which IMAs were used, corticosteroids have often been used as primary treatment of checkpoint inhibitor-induced AEs, with infliximab as secondary therapy in corticosteroid-refractory cases [30]. Our systematic review found that IMAs were used in approximately half of patients receiving the ipilimumab–nivolumab combination or ipilimumab alone (47–65%). However, only 15% of patients given nivolumab monotherapy were treated with IMAs [7,19]. It is difficult to determine whether more patients receiving any of the checkpoint inhibitor regimens should have been treated with IMAs, even when it is known how commonly AEs occurred, because rates of IMA use were not reported by AE grade (i.e., if most were grade 1, they would not require IMA treatment).

We did not find any studies that evaluated healthcare utilization or costs with combination checkpoint inhibitors. This finding is of concern, since patients experiencing grade 3–4 AEs with checkpoint inhibitor monotherapy can heavily utilize healthcare resources, including outpatient visits, medication(s), specialist visits and hospitalization [30–32]. Because inpatient and emergency care often represent some of the most expensive services provided by a payer, it is likely that the treatment costs for GI AEs would be offset by savings in reducing the use of these services. This would be notable in patients receiving combinations due to their much higher healthcare requirements for resulting AEs, particularly severe GI AEs that would be likely to drive patients to seek care at a hospital.

One of the most important reasons to resolve GI AEs in patients receiving checkpoint inhibitors, especially in those receiving combinations with their associated high AE rates, is to ensure that patients can remain on effective combination treatment. Checkpoint inhibitors have been a major advancement in the treatment of advanced melanoma, improving overall survival, PFS and ORR compared with other experimental and established treatment regimens [13,33,34]. The approval of combination ipilimumab plus nivolumab further increased these benefits [12]. Unfortunately, combining ipilimumab with nivolumab leads to higher discontinuation rates due to GI AEs compared with ipilimumab or nivolumab monotherapy [7,26]. Discontinuing treatment early or reducing therapy to single-agent nivolumab after a patient experiences AEs may reduce efficacy of therapy [12,13,33,34]. With better treatments for GI AEs resulting from combination checkpoint inhibitors, patients with advanced melanoma can remain on their combination treatment regimen, potentially leading to improved clinical outcomes.

This study had a number of limitations. First, GI AEs were only systematically evaluated for combination checkpoint inhibitors, not checkpoint inhibitor monotherapy. Many primary studies and systematic reviews have evaluated checkpoint inhibitor monotherapy, which motivated the current focus on combination therapy, with single-agent data only reported for comparison from monotherapy arms of combination therapy studies. A future systematic review might encompass both combination therapy and monotherapy for a more comprehensive evaluation of GI AEs, and may also allow meta-analysis of the data. Second, all identified studies of combination therapy only evaluated the combination of ipilimumab plus nivolumab. Therefore, conclusions regarding outcomes of combination use may only apply to this particular combination and not other combinations of checkpoint inhibitors. Third, the absence of studies evaluating the humanistic or economic burden of GI AEs limits a full understanding of the burden of these treatment effects, which might be used to inform cost–effectiveness of checkpoint inhibitors as well as the therapies used to treat their AEs.

## Conclusion & future perspective

GI AEs occurred at a higher rate and greater severity in patients treated with the ipilimumab–nivolumab combination versus monotherapy. These AEs promote discontinuation of combination therapy, which can undermine its superior efficacy to that of monotherapies. Novel treatments for AEs could improve clinical outcomes for patients who would benefit from combination therapy if they could tolerate and persist on it. The lack of research on economic and humanistic burden of GI AEs with combination checkpoint inhibitors for advanced melanoma represents an evidence gap that merits exploration in future studies.

**Executive summary**

**Incidence of gastrointestinal adverse events**

- Approximately half of patients treated with ipilimumab plus nivolumab experienced a treatment-related gastrointestinal (GI) adverse event (AE). Colitis and diarrhea were among the most common with the ipilimumab–nivolumab combination; diarrhea was more common, but colitis was more severe.

**Risk of GI AEs**

- The risk of developing any grade of diarrhea was higher with ipilimumab plus nivolumab compared with most individual checkpoint inhibitors or chemotherapy alone. However, combination therapy did not impart a higher risk of nausea, grade 3–5 diarrhea or grade 3–5 colitis.

**Timing & severity of AEs**

- GI AEs in patients receiving the combination may begin on average during the second month of therapy and can be severe enough to cause discontinuation in 15% of patients.

**Treatment of GI AEs**

- Most patients treated with ipilimumab plus nivolumab required immunomodulatory agents for the treatment of GI AEs, most of which resolved in just over a month.

**Economic & humanistic burden of GI AEs**

- This systematic search of the literature did not find any studies on the economic or humanistic burden associated with GI AEs with combination checkpoint inhibitors.

**Conclusion**

- The epidemiological and clinical burden of treatment-related GI AEs has been well studied for the ipilimumab–nivolumab combination, but not other combinations of checkpoint inhibitors. The economic and humanistic burden of AEs with combination checkpoint inhibitors remains largely unexamined.
- GI AEs occur in approximately half of patients with ipilimumab plus nivolumab and are often severe.
- The ipilimumab–nivolumab combination consistently produces more GI AEs than either agent used alone, especially nivolumab.
- Patients receiving the ipilimumab–nivolumab combination are more likely to discontinue due to GI AEs than those on monotherapy, undermining the efficacy of therapy. Therefore, better treatments for AEs may improve clinical outcomes for patients who may benefit from combination therapy.
